# Gender and Cutaneous Leishmaniasis in Israel

**DOI:** 10.3390/tropicalmed7080179

**Published:** 2022-08-12

**Authors:** Michal Solomon, Inbal Fuchs, Yael Glazer, Eli Schwartz

**Affiliations:** 1 Department of Dermatology, Chaim Sheba Medical Center, Tel Hashomer, The Sackler School of Medicine, Tel Aviv University, Tel Aviv 8436322, Israel; 2 Clalit Health Services-Southern District Department of Family Medicine, Faculty of Health Sciences, Ben Gurion University, Beer Sheva 84105, Israel; 3 Division of Epidemiology, Ministry of Health, Jerusalem 9462401, Israel; 4 Center for Geographic Medicine and Tropical Diseases, Chaim Sheba Medical Center, Tel Hashomer, The Sackler School of Medicine, Tel Aviv University, Tel Aviv 6997801, Israel

**Keywords:** cutaneous leishmaniasis, mucocutaneous leishmaniasis, *Leishmania major*, *Leishmania tropica*, *Leishmania braziliensis*, gender

## Abstract

Leishmaniasis is estimated to be more common in males than in females. Our purpose was to evaluate differences in preponderance in relation to sex and gender across cutaneous and mucocutaneous leishmaniasis in Israel. An observational study was performed, including cases of endemic CL (cutaneous leishmaniasis) in Israel, and imported MCL (mucocutaneous leishmaniasis). CL is a notifiable disease and is supposed to be reported to the Ministry of Health (MOH). The MOH database shows that males as more likely to be infected by leishmania, with an incidence of 5/100,000 in males vs. 3.5/100,000 in females. However, while conducting a demographic house-to-house survey in several locations in Israel where CL is highly endemic, among 608 people who were screened only 49% were males in *Leishmania major (L. major)* endemic regions and 41% were males in *Leishmania tropica* (*L. tropica*) endemic regions, while among 165 cases of imported New-World cutaneous leishmaniasis in Israeli travelers freturning from abroad, 142 (86%) were males. It may be postulated that there is no real gender difference in leishmanial infection, but, perhaps, infections are more commonly seen in men because of referral/reported bias, due to more risk-taking behaviors by men or, perhaps, men are less likely to strictly adhere to recommended preventive measures and thus increase their risk of contracting the disease.

## 1. Introduction

Cutaneous leishmaniasis (CL) is endemic to Israel and is caused mainly by Old-World cutaneous leishmaniasis (OWCL). In the past, CL in Israel was almost exclusively attributed to *Leishmania major (L. major)* [1,2]. The disease is typically more common in the Arava, along the Jordan River Valley and in the rural areas of the Negev Desert. However, over the last two decades or so there have been increasing reports of CL due to *Leishmania tropica* (*L. tropica*) in several other regions of Israel, including the hilly and mountainous regions, Samaria in the center of the country and in northern Israel [3,4].

Additionally, travel to Central and South America has increased over the past two decades, mainly among young Israeli adults, increasing potential exposure to various tropical diseases, including leishmaniasis [5]. New-World cutaneous leishmaniasis (NWCL), which is endemic to some parts of the Americas, is caused by *Leishmania viannia* and *Leishmania mexicana* species complexes. Infection with *L. viannia* species, particularly *Leishmania viannia braziliensis* (*L. (V) braziliensis*), results in cutaneous leishmaniasis (CL) which tends to be persistent and may be further complicated by mucocutaneous leishmaniasis (MCL) [6]. Among Israeli travelers *L*. *(V) braziliensis* was found to be the dominant species, acquired mostly in the Amazon region of Bolivia, causing CL and MCL [7].

Most studies of CL and MCL provide minimal data on sex and gender. Differences between the sexes in the incidence and severity of infection might be related to physiological or genetic constitutions [8], but could also result from differences in exposure, use of preventive strategies [9], participation in high-risk activities, attractiveness to vectors, routes of pathogen entry, or processing of pathogens and cellular responses. A recent study of sex-related differences in leishmaniasis, showed that environmental exposure and healthcare access [10] alone do not explain the variance. Moreover, transcriptomic evidence reveals that biological sex is a variable which impacts physiology, drug metabolism, immune response, and, consequently, the progression of disease [10].

In this paper, we sought to evaluate gender and sex differences among CL patients in Israel as well as returning Israeli travelers with CL/MCL.

## 2. Materials and Methods

Leishmaniasis is a notifiable disease in Israel. All cases reported to the Ministry of Health (MOH) were analyzed based on Leishmania species and gender. Because many cases are not reported, in certain areas where CL is highly endemic, we chose to carry out a demographic survey. One such region was in Southern Israel (Negev) where *L. major* is the common species, and where, starting in 2010, a dramatic increase in CL cases was observed by the primary clinic staff of three kibbutzim.

Clinical and demographic data of patients who presented to the three kibbutzim clinics were recorded. In the context of outbreak investigation, a questionnaire was sent to all kibbutz members including those who had not presented to the clinic, inquiring if any household members had cutaneous leishmanial lesions and did not present to the clinic. Demographic and clinical data concerning those additional patients were completed by phone interviews conducted by the kibbutz nurses.

The other region was in Samaria, in the central part of Israel, where *L. tropica* is the common species and where similar house-to-house enquiries were made.

In addition, we analyzed data from returning travelers with CL and MCL due to *L. (V) braziliensis* who presented at Sheba Medical Center, a tertiary medical center in Israel.

OWCL was diagnosed when patients from endemic areas developed cutaneous lesions (ulcers, nodules, or papules) that were clinically similar to leishmaniasis, when biopsy or smear specimens revealed Leishmania amastigotes within a dermal infiltrate, or when a polymerase chain reaction (PCR) assay revealed positive results for *L. major* or *L. tropica*.

NWCL was diagnosed when (i) cutaneous lesions (ulcers, nodules, or papules) that were clinically compatible with leishmaniasis were observed, and one or both of the following tests came back positive, (ii) a smear or biopsy specimen showed *Leishmania* amastigotes within a dermal or mucosal infiltrate, and (iii) a polymerase chain reaction (PCR) assay tested positive for *Leishmania vianna braziliensis*. [5]

MCL: All cases of MCL were symptomatic with either nasal or oral symptoms and proven by PCR, culture, or biopsy for leishmaniasis [11].

### Statistical Analysis

Data entry and analysis was performed using the statistical package for the social sciences (SPSS) version 23.0 for Windows software. Interquartile range (IQR) and the median were used to express continuous variables, whereas percentages were used to indicate categorical variables. Fisher’s exact test (two-tailed) was used to compute the *p*-value in the prevalence assessment. A *p*-value < 0.05 was considered significant.

## 3. Results

The nationally reported cases of CL in the MOH registry showed 382 cases of *L. major* during the years 2012–2016, among them 203 (53%) males and 179 (47%) females. There were 348 cases of *L. tropica*, among them 191 (55%) males and 157 (45%) females

The MOH database shows that males are also more likely to be infected with leishmania, with a male incidence of 5/100,000 vs. a female incidence of 3.5/100,000 in 2015.

In our house-to-house survey, among the total population of three Kibbutzim in the Negev in southern Israel—which is known to be an endemic area for *L. major*—1146 people were screened, and 330 patients with *L. major* CL were identified between the years 2010 and 2013; 164/330 (49.6%) were males. Demographic data are presented in Table 1. The median age at diagnosis was 48 years (range 0.6–87 years).

In Peduel, a village in Samaria, in central Israel, an endemic area for *L. tropica,* between the years 2008 and 2012; 1096 people (25% of the total village population) were screened. Among them, 278 (25.3%) were diagnosed with CL. Gender distribution was 114 (41%) males and 164 (59%) females in comparison to 49% males in the *L. major* group (*p* value = 0.03).

Altogether, in these population-based screenings of four villages, 278 males (45.7%) and 330 females (54.2%) were diagnosed. Most of the patients in the *L. major* group were between the ages of 15 and 64 years (65%), however in the *L. tropica* group most of the patients were children between the ages of 0 and 15 years (51.4%) (Table 1).

Among travelers, the total number of patients diagnosed with NWCL from South America at Sheba Medical Center during the years 1993–2021 was 165. All the cases were acquired in Bolivia’s Amazon region, where *Leishmania (V.) braziliensis* is known to be endemic. In this cohort, the majority were males 140 (85%).

MCL: Nineteen patients (11.5%) were diagnosed with MCL among the cohort of travelers returning from Latin America with leishmaniasis. Among them the majority (18 patients (95%)) were also males. The high rate of males in the MCL group was non-significantly higher than that of the other NWCL cases (95% vs. 84%) (Table 2).

## 4. Discussion

Gender relates to a person’s self-representation as male or female and to the way that social institutions influence that person, and it is shaped by environment and experience [9], while sex is biologically determined (the sexual genotype is XX in the female and XY in the male). Most studies of NWCL and MCL provide scant information regarding gender and sex in relation to leishmania infection. Disparities between the sexes in the incidence and severity of infection might be attributed to ‘sex’ differences such as genetic and physiological constitutions [8], or to ‘gender’/cultural differences such as participation in high-risk activities [9].

In this study we examined three cohorts of leishmanial patients regarding their gender distribution. Each cohort gave a different result. The most striking difference between males and females was found among travelers returning from South America where NWCL was acquired.

There is epidemiological evidence of a sex bias in NWCL [12]. Data from the New World documented a higher incidence of CL in males than in females in Mexico and Colombia, and similar studies from all around Brazil reveal males developing cutaneous manifestations of leishmaniasis more frequently than females [12,13,14,15,16,17,18]. A nationwide study found that in infants under the age of one year, males had a higher rate of CL in comparison to females. Minipuberty, a transient postnatal rise in sex steroid levels that exhibits clear hormonal variations between boys and girls has been linked to other sex-biased infectious diseases [19,20] and may be the cause of the predominance in male infants. Overall, the prevalence of CL among males increased at puberty, peaked during adulthood, and then decreased in the elderly. This occurred even though it is unlikely that males experience increased parasite exposure, suggesting that the observed sex dichotomy has a biological basis [19,21]. Together, these data lend credence to the hypothesis that innate biological characteristics place males at higher risk of New-World CL.

Reported leishmaniasis cases in travelers show that most cases are in males [22]. The rate of infected males among returning Israeli travelers was 84% of all those diagnosed with NWCL. This high proportion of males is far greater than the known distribution of Israelis visiting the tropics, which is about 50% of each gender [23]. However, all these infections were contracted while on adventure trips in the Amazon region in Bolivia, an area known to be endemic for *Leishmania (V.) braziliensis* [22,24]. We therefore cannot rule out the possibility that female travelers are less likely to visit this region specifically or that they take stricter precautions. Indeed, the Geosentinel report about NWCL also reported a high rate of male travelers (72%) [22]. The additional observation we made among these patients was assessing the rate of mucosal leishmania among those who contracted NWCL. Among the cohort mentioned above, 11% (19/165) of travelers developed MCL. Although the rate of MCL was higher in males (12%) vs. 4% in females, this was a non-significant difference (Table 2). A higher rate of MCL in males was also documented in the local population [24]. Whether the lack of a clear gender difference regarding this severe complication in our cohort is due to the small sample size or due to a real lack of difference should be further investigated.

Among OWCL we presented two cohorts; one was the reported cases to the MOH while the other was the house-to-house survey.

Israel is endemic for CL and all cases must be reported to the Ministry of Health (MOH), whose database also shows males as more prone to leishmania infection, with an incidence of 5/100,000 in males vs. 3.5/100,000 in females. We chose to carry out a demographic survey in several areas where CL is highly endemic because it is an established fact that there is significant under-reporting of the condition. One of these regions was in Southern Israel (Negev) where *L. major* is the common species, while the other was in Central Israel where the common species is *L. tropica*. A total of 608 people was screened, and results showed that altogether there were 278 males (45.7%) and 330 females (54.2%) infected. However, in the *L. major* endemic region, 49% were males and 51% were females, while in the *L. tropica* endemic region 41% were males and 59% were females (Table 1).

Multiple endemic regions of the Old World have documented CL incidence varying between the sexes. It is argued that whether there is a sex bias, and its direction if there is, is determined by a complex interaction between environmental, host gender, and biological factors, and the infective Leishmania species [25,26,27,28,29]. Our data clearly demonstrate that the rate of infection is relatively comparable across males and females when population-based screening is carried out, avoiding referral and reporting biases, and or/risky travel to endemic regions.

Patient and animal models of infection of Old-World species have both shown sex-based differences [30]. It was estimated that CL due to *L. major* is more common in males [28,31], while in some endemic areas *L. tropica* infection seems to be more prevalent among females, and that males are more likely to develop viscerotropic leishmaniasis [10,27,32]. However, the mechanisms responsible for this variability are elusive [30].

Although in animal models sex was found to be a risk factor, we did not find it in our results. It was estimated that leishmaniasis is more common in males. However, in our cohort when house-to-house screening was done, the rate of infection in males and females was comparable. The reason for this difference is the avoidance of referral and reporting biases, or/risky travel to endemic regions.

## 5. Conclusions

In order to check the rate of CL and MCL infections, house-to-house screening is the preferred method. Our research in Israel demonstrates that there are no real differences between the sexes in cutaneous leishmaniasis infection rate, but infections are more frequently seen in men, possibly due to reporting and referral bias, riskier behavior, and travel to more remote destinations where the risk of contracting tropical diseases is higher. Men may also be less likely to adhere to preventive measures, which increases their risk of contracting the disease.

## Figures and Tables

**Table 1:** *CL* in three kibbutzim and one village endemic for Leishmania in Israel.

	*L. major*				*L. tropica*	Total Old-World Leishmaniasis (*L. major* and *L. tropica*)
	Kibbutz 1	Kibbutz 2	Kibbutz 3	Total	Village (Sample)	
Cases/total population (%)	160/346 (46.2)	148/380 (39)	22/420 (5.2)	330/1146 (28.7)	278/1096 (25.3)	608
Gender						
Male *	78 (48.8)	75 (50.7)	11 (50)	164 (49.6)	114 (41)	278 (45.7)
Female	82 (51.2)	73 (49.3)	11 (50)	166 (50.3)	164 (59)	330 (54.2)
Age						
0–15	10 (6.2)	19 (12.8)	3 (13.6)	32 (9.6)	143 (51.4)	176
15–64	98 (61.2)	101 (68.2)	16 (72.7)	215 (65.1)	134 (48.2)	351
65+	52 (32.5)	28 (18.9)	3 (13.6)	83 (25.1)	1 (0.4)	85

* p-value between males and females is non-significant. *L. major* = *Leishmania major* and *L. tropica* = *Leishmania tropica*.

**Table 2:** Comparison between New-World CL patients and MCL patients with cutaneous leishmaniasis and mucocutaneous leishmaniasis.

	Total New-World Leishmania Cases	Cutaneous LeishmaniAsis	Mucocutaneous LeishManiasis	Percentage of Mucocutaneous Leishmaniasis *
No. of patients	165	146	19	11.5%
Males	142	124 (84%)	18 (95%)	12.6%
Females	23	22 (15%)	1 (5%)	4.3%
Mean age (years)		24.2	27.6	

* *p* value is insignificance. CL, cutaneous leishmaniasis and MCL, mucocutaneous leishmaniasis.

## Data Availability

No new data were created or analyzed in this study. Data sharing is not applicable to this article.

## Abbreviations

NWCL	New-World cutaneous leishmaniasis
OWCL	Old-World cutaneous leishmaniasis
*L. (V.) braziliensis*	*Leishmania viannia braziliensis*
*L. Major*	*Leishmania major*
*L. Tropica*	*Leishmania tropica*
CL	Cutaneous leishmaniasis
MCL	Mucocutaneous leishmaniasis
PCR	Polymerase chain reaction
